# The complete chloroplast genome of the multipurpose and traditional herb, *Ruta graveolens* L.

**DOI:** 10.1080/23802359.2019.1678426

**Published:** 2019-10-18

**Authors:** Li-Zhen Ling, Shu-Dong Zhang

**Affiliations:** School of Biological Sciences and Technology, Liupanshui Normal University, Liupanshui, China

**Keywords:** Chloroplast genome, phylogenetic analysis, *Ruta graveolens*, Rutaceae

## Abstract

*Ruta graveolens* L. is a perennial plant belonging to the family Rutaceae and has been used for traditional medicines for a long time. In this study, the complete chloroplast (cp) genome sequence of *R. graveolens* was first reported and characterized. The cp genome is 157,434 bp in length and contains a pair of inverted repeats (IRs, 26,868 bp) separated by a large (85,387 bp) and small (18,311 bp) single-copy regions. A total of 132 genes were predicted, including 87 protein-coding genes, 37 tRNA genes and 8 rRNA genes. The phylogenetic analysis suggested that *R. graveolens* is more closely related to the monophyletic subfam. Aurantioideae.

*Ruta graveolens* L. is a perennial herb (Rutoideae, Rutaceae), which is native to the Mediterranean region of southern Europe and northern Africa and now cultivated in many countries of the world (Miguel 2003). Since ancient times, *R. graveolens* has been used in traditional medicines for the relief of pain, eye problems, rheumatism and dermatitis. Recently, *R. graveolens* has shown various pharmacological activities, including contraceptive, hypotensive, antimicrobial, analgesic, anti-inflammatory, antipyretic, antidiabetic and insecticidal activities (Miguel 2003; Salvo et al. 2010; Asgarpanah 2012; Kannan and Babu 2012; Parray et al. 2012; Malik et al. 2016). At now, more than 120 natural compounds mainly including acridone alkaloids, coumarins, essential oils, flavonoides, and fluoroquinolones have been found in the roots and aerial parts (Asgarpanah 2012; Kannan and Babu 2012; Parray et al. 2012; Malik et al. 2016). To provide genomic resources for investigating the evolution of *R. graveolens*, the complete chloroplast (cp) genome of this species was analyzed from high-throughput Illumina sequencing reads.

The fresh leaves of *R. graveolens* were collected from Kunming Institute of Botany, CAS (N25°08′21″, E102°44′30″, 1,950 m) and the specimen (lpssy0212) was deposited at the herbarium of the Liupanshui Normal University (LPSNU). The genomic DNA was extracted and sequenced as previously described (Zhang et al. 2019). Approximately 2 Gb raw data were generated with pair-end 150 bp read length on the Illumina HiSeq 4000 Platform. The cp genome of *R. graveolens* was *de novo* assembled using the GetOrganelle pipeline (https://github.com/Kinggerm/GetOrganelle) and all genes were annotated using Dual Organellar Genome Annotator (DOGMA) (Wyman et al. 2004). The complete cp genome sequence of *R. graveolens* was deposited in GenBank database (accession no. MN326012).

The complete *R. graveolens* cp genome is 157,434 bp in length, including a large single copy (LSC) region of 85,387 bp, a small single copy (SSC) region of 18,311 bp, and a pair of inverted repeats (IRs) of 26,868 bp each. The cp genome shows the GC content of 37.2% and contains 132 genes, including 87 protein-coding genes, 37 transfer RNA (tRNA) genes, and 8 ribosomal RNA (rRNA) genes. Of them, 15 distinct (*atpF*, *ndhA*, *ndhB*, *petB*, *petD*, *rpl16*, *rpl2*, *rpoC1*, *rps16, trnA-UGC*, *trnG-GCC*, *trnI-GAU*, *trnK-UUU*, *trnL-UAA* and *trnV-UAC*) contain one intron and three genes (*clpP, rps12* and *ycf3*) have two introns.

The Rutaceae family comprises 150–170 genera and approximately 2,040 species and includes four subfamilies: Aurantioideae, Cneoroideae, Rutoideae, and Amyridoideae (Morton and Telmer 2014). The cp genomes of *R. graveolens* and previously published species of Rutaceae were used for phylogenetic analysis. In this study, the cp genomes from 24 representative species from two subfamiles (Aurantioideae and Amyridoideae) were downloaded and their GenBank accession numbers are provided in Figure 1. Two species (*Leitneria floridana* and *Ailanthus altissima*) from Simaroubaceae were used as the outgroups. Figure 1 shows that there is very strong support for a monophyletic subfam. Aurantioideae, which is consistent with the previous results based on the cpDNA and nuclear DNA sequences (Groppo et al. 2008; Bayer et al. 2009; Morton and Telmer 2014; Appelhans et al. 2016). *Ruta graveolens* (Rutoideae) is sister to Aurantioideae, which are strongly supported as sister to *Phellodendron* and *Zanthoxylum* (Amyridoideae) (Figure 1).

**Figure 1.**
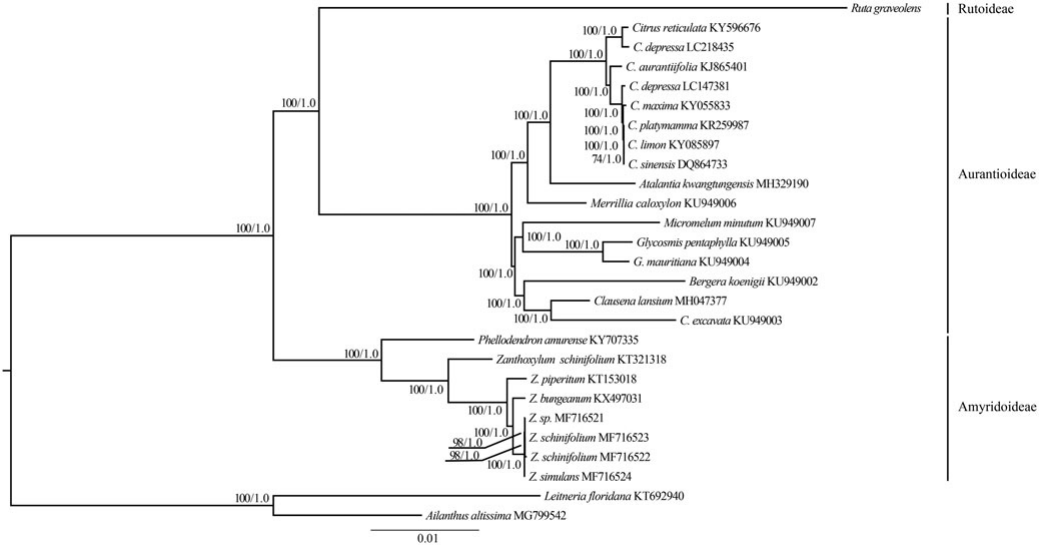
The maximum likelihood (ML) tree of 25 species from three subfamilies of Rutaceae inferred from the complete chloroplast genome sequences. Numbers at nodes correspond to ML bootstrap percentages (1000 replicates) and Bayesian inference (BI) posterior probabilities.
